# Association of Autophagy-Related Gene Expression Profiles with Survival in Diffuse Astrocytic Tumors

**DOI:** 10.3390/cancers18081215

**Published:** 2026-04-10

**Authors:** İlker Kiraz, Gözde Topel, Veli Kaan Aydın, Serkan Civlan, Ümit Akın Dere, Mehmet Erdal Coşkun, Nagihan Yalçın, Gergana Lengerova, Martina Bozhkova, Steliyan Petrov, Aylin Köseler

**Affiliations:** 1 Department of Neurosurgery, Faculty of Medicine, Pamukkale University, 20160 Denizli, Türkiye; ikiraz@pau.edu.tr (İ.K.); scivlan@pau.edu.tr (S.C.); udere@pau.edu.tr (Ü.A.D.); mecoskun@pau.edu.tr (M.E.C.); 2 Department of Pathology, Faculty of Medicine, Pamukkale University, 20160 Denizli, Türkiye; gtopel@pau.edu.tr (G.T.); nyalcin@pau.edu.tr (N.Y.); 3 Department of Biophysics, Faculty of Medicine, Pamukkale University, 20160 Denizli, Türkiye; vkaydin@pau.edu.tr; 4 Department of Medical Microbiology and Immunology “Prof. Dr. Elissay Yanev”, Medical University of Plovdiv, 4002 Plovdiv, Bulgaria; gergana.lengerova@mu-plovdiv.bg (G.L.); martina.bozhkova@mu-plovdiv.bg (M.B.); steliyan.petrov@mu-plovdiv.bg (S.P.); 5 Research Institute, Medical University of Plovdiv, 4002 Plovdiv, Bulgaria

**Keywords:** glioma, autophagy, SQSTM, Beclin1, Atg5, Atg7

## Abstract

**Background**: The aim of this study is to investigate the relationship between the expression levels of autophagy-related genes (SQSTM1, Beclin1, Atg5, and Atg7) in diffuse astrocytic tumors and clinicopathological parameters, including tumor grade, IDH mutation status, and survival outcomes. **Materials and Methods:** A total of 150 histopathologically confirmed diffuse astrocytic tumor cases were retrospectively analyzed. Clinical data were extracted from patient records. Gene expression levels were determined using qRT-PCR and evaluated by the 2^−ΔCt^ method, where lower ΔCt values indicate higher gene expression. IDH1 R132H mutation status was evaluated by immunohistochemistry. **Results:** No statistically significant differences were observed in the expression levels of SQSTM1, Beclin1, Atg5, and Atg7 across WHO tumor grades (*p* > 0.05). However, when analyzed by IDH status, IDH-mutant tumors exhibited significantly higher gene expression levels (demonstrated by lower ΔCt values) of Beclin1 (*p* = 0.046) and Atg5 (*p* = 0.027) compared to IDH wild-type tumors. In multivariate Cox regression analysis, age and WHO tumor grades were confirmed as independent prognostic factors. Crucially, higher SQSTM1 expression independently predicted worse clinical outcomes, specifically poorer overall survival (OS) (*p* = 0.004) and shorter progression-free survival (PFS) (*p* = 0.031). Additionally, elevated Beclin1 expression was identified as an independent predictor of worse OS (*p* = 0.023). **Conclusions**: This study demonstrates that increased expression of autophagy-related genes, particularly SQSTM1 and Beclin1, serves as a robust indicator of poor prognosis and shorter survival times in diffuse astrocytic tumors. Furthermore, the elevated expression of Beclin1 and Atg5 in IDH-mutant cases highlights a complex metabolic interplay that warrants further investigation as potential therapeutic targets.

## Simple Summary

Brain tumors known as gliomas are highly aggressive and difficult to treat. A key reason for this is a cellular recycling process called autophagy, which helps tumor cells survive under harsh conditions and resist therapies. In this study, we aimed to understand how the genes controlling this recycling process affect patient survival and tumor characteristics. By analyzing tumor samples from 150 patients and validating our findings using a large, independent public database, we measured the activity of four specific autophagy genes. Our results revealed that the elevated activity of certain genes, particularly SQSTM1 and Beclin1, is closely linked to shorter patient survival times and specific tumor mutations (such as the IDH mutation). These findings provide valuable insights for the research community, suggesting that autophagy genes could serve as biological markers to better predict patient outcomes. Ultimately, understanding and targeting this recycling mechanism could pave the way for developing more effective, targeted treatment strategies for brain tumor patients.

## 1. Introduction

Glioblastoma is defined as the most aggressive and highly lethal primary brain malignancy in adults [1]. According to the 2021 WHO Classification of Tumors of the Central Nervous System, glioblastomas are specifically defined as grade 4, IDH-wildtype diffuse astrocytic tumors [2]. One of the mechanisms determining tumor biology is autophagy. This cellular process, through degradation and recycling of proteins and organelles, maintains cellular homeostasis; however, it plays a complex role in tumor development and treatment [3]. Beyond intracellular homeostasis, autophagy is pivotal in the pathogenesis of various diseases, including neurodegenerative disorders and cancer [4].

Dysregulation of autophagy has been highlighted as a key factor affecting tumor survival and response to therapy in high-grade gliomas such as glioblastoma [5]. To investigate the mechanics of autophagosome formation, Atg5 and Atg7 were specifically selected for this study. These genes act as indispensable, core components of the ubiquitin-like conjugation systems strictly required for autophagic vesicle elongation [6,7]. While both are critical nodes in autophagic flux, evaluating them simultaneously allows us to determine whether the entire elongation complex is uniformly regulated or if specific components (such as Atg5) are differentially targeted under tumor-specific metabolic stress conditions.

The median survival for glioblastoma patients remains approximately one year, making it essential to understand how autophagy-related gene expression affects glioma development and progression [8]. Within this scope, transcriptomic differences between low-grade gliomas and normal brain tissues have revealed the differential expression of 35 autophagy-related genes [9]. This scenario indicates the complex role of autophagy in glioma pathogenesis and its bidirectional effects at different tumor grades, necessitating extensive study [10].

The expression of autophagy-related proteins such as LC3, Beclin 1, and p62 is more frequently elevated in high-grade gliomas and has been linked to poor survival [11]. Conversely, some studies suggest autophagy plays a tumor-suppressive role, with more sustainable activity observed in low-grade gliomas [12]. Isocitrate dehydrogenase (IDH) mutations, particularly IDH1 R132H, are fundamental to the molecular classification of diffuse gliomas. Clinically, IDH-mutant tumors are generally associated with a significantly better prognosis and longer survival compared to IDH-wildtype cases. However, these mutations induce profound metabolic reprogramming, primarily through the accumulation of the oncometabolite 2-hydroxyglutarate (2-HG) [13,14].

Interestingly, recent evidence suggests that 2-HG can paradoxically induce autophagy pathways. While IDH-mutant tumors have an overall better prognosis, the elevated activation of autophagy-initiating genes under 2-HG-induced metabolic stress may serve as an adaptive survival mechanism for these specific cells [15]. Therefore, understanding whether this heightened autophagic state in IDH-mutant gliomas correlates with improved or worsened survival within specific cohorts remains a critical clinical question.

Within this complex network, Beclin1 serves as a crucial molecular rheostat. Beyond its primary role in initiating autophagy via the PI3K complex, Beclin1 heavily interacts with anti-apoptotic proteins of the Bcl-2 family. The dynamic disruption or promotion of the Beclin1/Bcl-2 complex dictates whether a tumor cell adapts to stress through protective autophagy or undergoes apoptotic cell death, underscoring its dual functional role [16,17].

High expression of p62 (SQSTM1) protein promotes glioblastoma progression, increasing cell proliferation, migration, glycolysis, and temozolomide resistance [18]. By interacting with LC3 and promoting its localization to autophagosomes, p62 is a major regulatory factor in glioblastoma stem cell migration and invasion [19]. Elevated levels of p62 and LC3B proteins have been linked to advanced tumor stages, poorer progression-free survival, and overall survival [20]. These findings highlight the significance of p62 and LC3B as prognostic and potential therapeutic markers [11].

In this study, we aimed to comprehensively investigate the relationship between expression levels of autophagy-related genes in glioma cases and clinicopathological parameters, including tumor grade, IDH mutation status, and survival outcomes. Autophagy has the potential to increase therapy efficacy by reducing chemotherapy resistance in tumors like glioblastoma [21], and molecules like p62 play an important role in tumor progression [22,23]. Therefore, a detailed analysis of the expression profiles of these genes is of great importance for the development of new prognostic markers and therapeutic strategies.

## 2. Materials and Methods

### 2.1. The Study Population

In this retrospective cohort study, we evaluated 150 patients surgically treated for diffuse astrocytic tumors at our institution. To ensure a homogenous and reliable study cohort, specific inclusion and exclusion criteria were established. Inclusion criteria were: (1) histopathologically confirmed primary diffuse astrocytic tumor (astrocytoma or glioblastoma), (2) availability of adequate formalin-fixed, paraffin-embedded (FFPE) tumor tissue, and (3) complete clinical follow-up and survival data. Exclusion criteria included: (1) patients diagnosed with non-astrocytic or mixed gliomas, (2) insufficient RNA quantity or quality for molecular downstream analysis, and (3) patients who received neoadjuvant chemotherapy or radiotherapy prior to surgical tumor resection.

All cases were independently re-evaluated by two experienced neuropathologists and graded according to the 2021 World Health Organization (WHO) Classification of Central Nervous System Tumors. The baseline demographic and clinicopathological characteristics of the study cohort are summarized in Table 1.

This study was approved by the Pamukkale University Faculty of Medicine Clinical Research Ethics Committee (Decision No: E-60116787-020-836824, Date: 25 February 2026). The study was conducted in accordance with the principles of the Declaration of Helsinki. Due to the retrospective design and use of archived material, the ethics committee waived the requirement for informed consent. All patient data were strictly anonymized prior to analysis.

### 2.2. Molecular Analysis

Molecular analyses were performed on FFPE tumor tissues. Serial sections (5–10 µm thick) were obtained from paraffin blocks. Tissues were deparaffinized using xylene and rehydrated through a descending graded ethanol series. Total RNA was isolated using a commercial RNA extraction kit optimized for FFPE tissues (RNeasy FFPE Kit, Qiagen, Hilden, Germany) according to the manufacturer’s protocol. RNA concentration and purity were assessed using a NanoDrop 2000 Spectrophotometer (Thermo Fisher Scientific, Wilmington, DE, USA) by evaluating the 260/280 nm absorbance ratio. Following quality control, complementary DNA (cDNA) synthesis was performed using a reverse transcriptase-containing kit (Qiagen, Hilden, Germany). The expression levels of autophagy-related genes (SQSTM1, Beclin1, Atg5, and Atg7) were quantitatively analyzed via real-time quantitative polymerase chain reaction (qRT-PCR). Amplification reactions were prepared in a 20 µL total volume using PowerUp SYBR Green Master Mix (Thermo Fisher Scientific, USA). The reactions were run on a CFX96 Real-Time PCR System (Bio-Rad, Hercules, CA, USA). The PCR protocol consisted of an initial denaturation at 95 °C for 10 min, followed by 40 cycles of denaturation (95 °C, 15 s), annealing (60 °C, 30 s), and extension (72 °C, 30 s). Amplification specificity was confirmed via melting curve analysis. All samples were analyzed in duplicate. Gene expression levels were normalized against the reference gene GAPDH, and relative expression was calculated using the 2^−ΔCt^ method. Primer sequences are detailed in Table 2 levels were calculated by the 2^−ΔCt^ method.

### 2.3. IDH Mutation Analysis

The IDH1 R132H mutation status was evaluated immunohistochemically. FFPE tissue sections were processed using standard staining protocols. A monoclonal antibody specific for the IDH1 R132H mutation (clone H09, Dianova GmbH, Hamburg, Germany; 1:20–1:100 dilution) was utilized. Antigen retrieval was performed via heat-induced epitope retrieval (HIER) in an appropriate buffer. Positive and negative controls were included in all runs. Cytoplasmic staining of tumor cells was deemed positive for the IDH1 mutation. Staining results were independently evaluated by two pathologists, with any discrepancies resolved by consensus.

### 2.4. Statistical Analysis

Statistical analyses were conducted using IBM SPSS Statistics Version 26.0 (IBM Corp., Armonk, NY, USA). The normality of continuous variables was assessed using the Kolmogorov-Smirnov and Shapiro-Wilk tests. Normally distributed data were presented as mean ± standard deviation (SD), while non-normally distributed data were presented as median and interquartile range (IQR). Categorical variables were reported as frequencies (n) and percentages (%). Relative gene expression levels (ΔCt values) across WHO tumor grades and IDH statuses were compared using the Mann-Whitney U test (for two groups) or the Kruskal-Wallis test (for three or more groups), followed by Dunn’s post-hoc analysis when appropriate. Progression-free survival (PFS) was defined as the time from diagnosis to disease progression or the last follow-up. Overall survival (OS) was defined as the time from diagnosis to death or the last follow-up. For survival analyses, the cohort was dichotomized into “High Expression” and “Low Expression” groups for each targeted gene based on the median ΔCt cut-off value. Survival curves were estimated using the Kaplan-Meier method, and differences between groups were assessed via the log-rank test. Univariate and multivariate Cox proportional hazards regression models were constructed to evaluate the independent prognostic value of clinical and molecular variables. Hazard ratios (HR) and 95% confidence intervals (CI) were calculated. All *p*-values were two-sided, with *p* < 0.05 considered statistically significant.

## 3. Results

### 3.1. Clinical and Histopathological Characteristics of the Study Cohort

**Figure 1:**
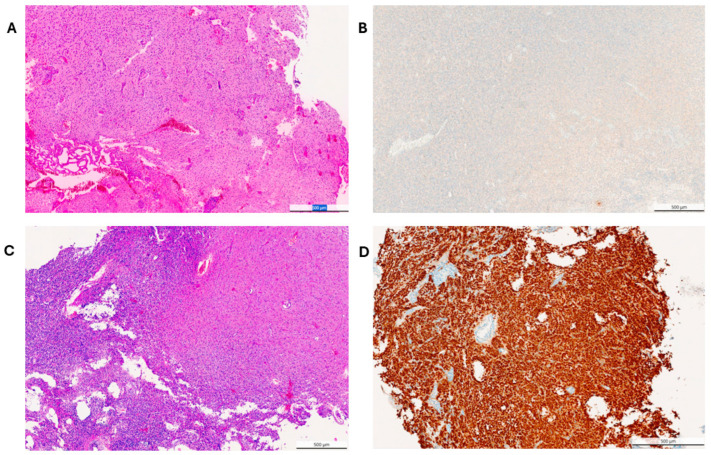
Representative histopathological and immunohistochemical findings of glioma cases included in the study. (**A**) Hematoxylin–eosin (H&E) staining of glioblastoma demonstrating increased cellularity and infiltrative tumor growth (original magnification ×20). (**B**) Immunohistochemical staining for IDH1 R132H mutation in glioblastoma showing cytoplasmic positivity in tumor cells (original magnification ×20). (**C**) Hematoxylin–eosin (H&E) staining of oligodendroglioma displaying characteristic infiltrative tumor morphology (original magnification ×20). (**D**) Immunohistochemical staining for IDH1 R132H mutation in oligodendroglioma demonstrating strong cytoplasmic immunoreactivity in tumor cells (original magnification ×20). Scale bar: 500 μm.

**Figure 2:**
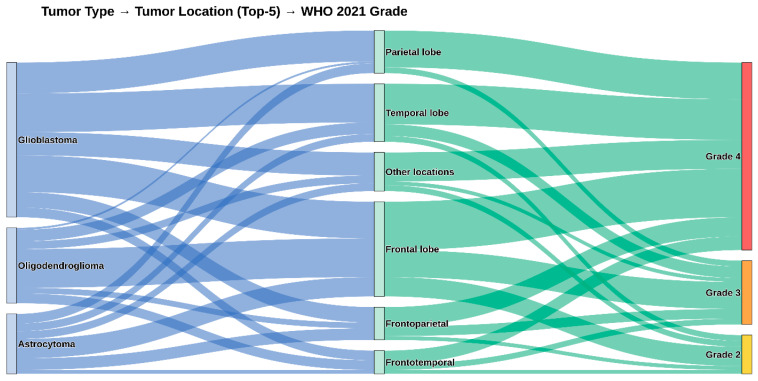
Sankey diagram shows the relationship between tumor type, tumor location (top five locations), and WHO 2021 grade in diffuse astrocytic tumors.

The study cohort comprised 150 patients surgically treated for diffuse astrocytic tumors. Comprehensive baseline demographics, including WHO 2021 classifications and IDH1 R132H mutation status, are detailed in Table 1. Representative histopathological and immunohistochemical findings of the glioma cases are shown in Figure 1. The complex distribution of tumor types according to anatomical location and WHO 2021 grade is illustrated in the Sankey diagram (Figure 2).

### 3.2. Autophagy Gene Expression According to WHO Tumor Grades

**Figure 3:**
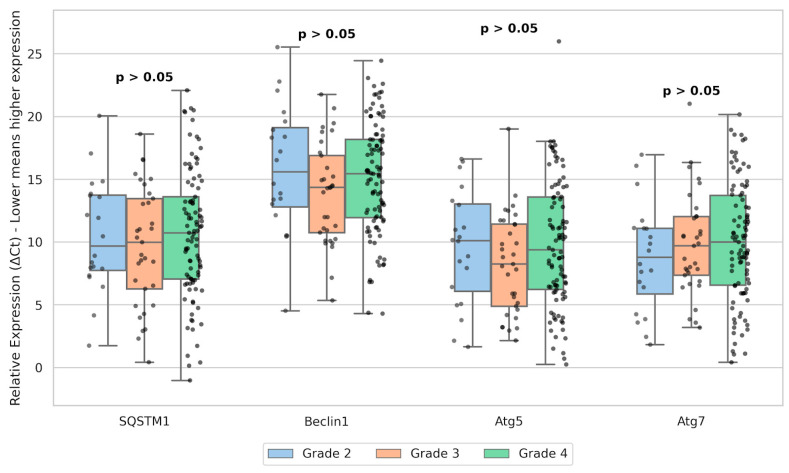
Boxplot showing the distribution of autophagy-related gene expression (SQSTM1, Beclin1, Atg5, and Atg7) across WHO 2021 tumor grades (G2–G4). Expression levels are presented as ΔCt values.

Quantitative analysis revealed no statistically significant differences in the relative expression levels of SQSTM1, Beclin1, Atg5, and Atg7 across WHO tumor grades (Grade 2, 3, and 4) (Kruskal-Wallis test, *p* > 0.05 for all target genes; Figure 3).

### 3.3. Autophagy Gene Expression by IDH Mutation Status

**Figure 4:**
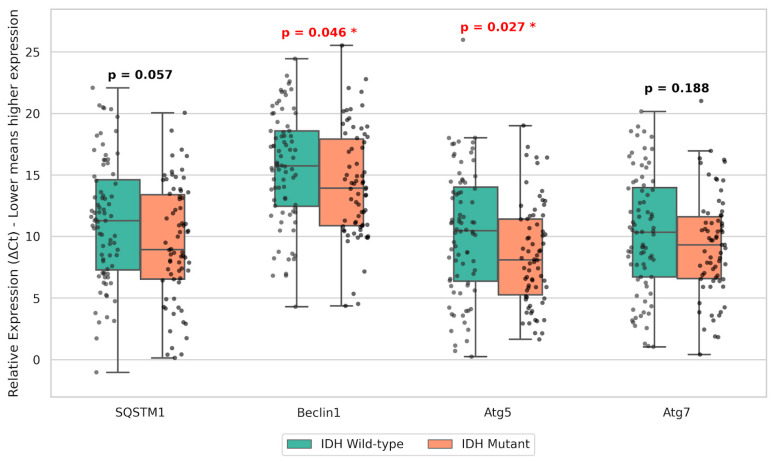
Boxplot showing the comparison of autophagy-related gene expressions (SQSTM1, Beclin1, Atg5, and Atg7) between IDH-mutant and IDH wild-type tumors. Expression levels are presented as ΔCt values, where lower ΔCt values indicate higher gene expression. Asterisks (*) indicate significant differences (*p* < 0.05) in gene expression levels between IDH-mutant and IDH wild-type groups.

When stratified by IDH1 R132H mutation status, significant differential expression was observed for specific autophagy initiators. IDH-mutant tumors exhibited significantly higher gene expression levels—demonstrated by significantly lower ΔCt values—of Beclin1 (*p* = 0.046) and Atg5 (*p* = 0.027) compared to IDH wild-type tumors. In contrast, the expression levels of SQSTM1 and Atg7 did not differ significantly between the IDH-mutant and wild-type cohorts (*p* > 0.05; Figure 4).

### 3.4. Survival Analysis

The median overall survival (OS) for the entire cohort was 9 months (IQR: 4–24), and the median progression-free survival (PFS) was 6 months (IQR: 3–16). In the multivariate Cox proportional hazards regression model, patient age and WHO tumor grade were confirmed as robust independent prognostic factors for both OS and PFS (*p* < 0.001).

Patients were dichotomized into “High Expression” (red solid lines) and “Low Expression” (blue dashed lines) groups based on median ΔCt cut-off values, where lower ΔCt values indicate higher gene expression. (A) Elevated SQSTM1 expression is significantly associated with worse overall survival outcomes (Log-rank *p* = 0.004). (B) Similarly, high Beclin1 expression independently predicts a significantly shorter overall survival time compared to the low expression cohort (Log-rank *p* = 0.023). The tables below each plot indicate the number of patients at risk at 6-month intervals.

**Figure 5:**
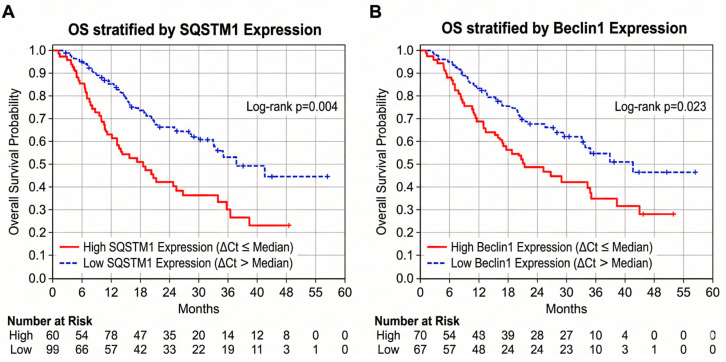
Kaplan-Meier overall survival (OS) curves for patients with diffuse astrocytic tumors, stratified by the relative expression levels of targeted autophagy genes. Patients were dichotomized into "High Expression" (red solid lines) and "Low Expression" (blue dashed lines) groups based on median ΔCt cut-off values, where lower ΔCt values indicate higher gene expression. (**A**) Elevated *SQSTM1* expression is significantly associated with worse overall survival outcomes (Log-rank *p* = 0.004). (**B**) Similarly, high *Beclin1* expression independently predicts a significantly shorter overall survival time compared to the low expression cohort (Log-rank *p* = 0.023). The tables below each plot indicate the number of patients at risk at 6-month intervals.

**Figure 6:**
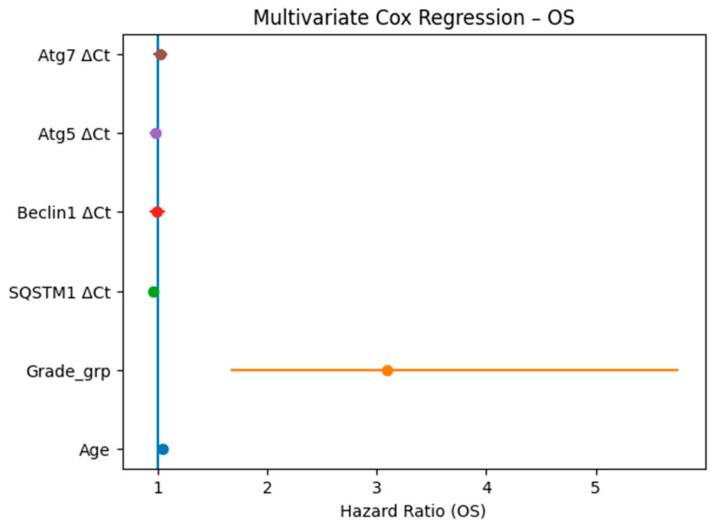
Multivariate Cox proportional hazards analysis showing the association of clinicopathological variables and autophagy-related gene expression levels with overall survival (OS).

**Figure 7:**
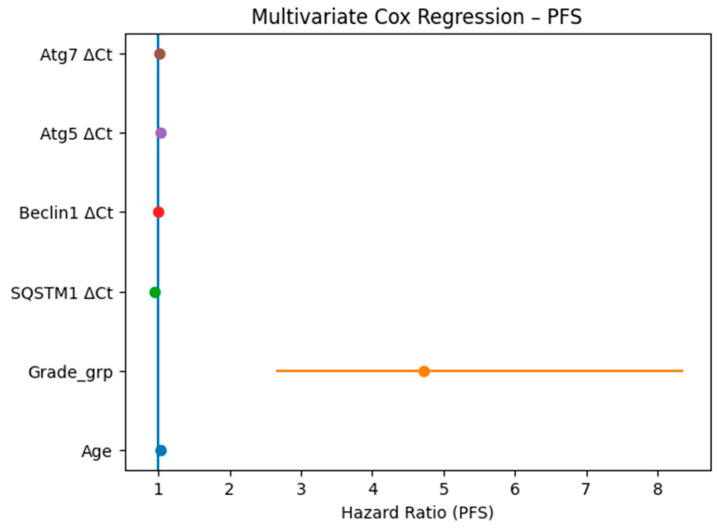
Multivariate Cox proportional hazards analysis showing the association of clinicopathological variables and autophagy-related gene expression levels with progression-free survival (PFS).

Crucially, survival analysis stratified by gene expression (using median ΔCt cut-offs) revealed that elevated expression of specific autophagy markers is strongly associated with worse clinical outcomes. High SQSTM1 expression independently predicted a significantly increased risk of death (worse OS, *p* = 0.004) and rapid disease progression (shorter PFS, *p* = 0.031). Similarly, elevated Beclin1 expression demonstrated a significant independent association with poorer OS (*p* = 0.023). Figure 5 illustrates the Kaplan-Meier survival curves, visually corroborating that patients with high SQSTM1 (Figure 5A) and Beclin1 (Figure 5B) expression experience significantly shorter overall survival times compared to their low-expression counterparts. Conversely, Atg5 and Atg7 expression levels did not show significant independent associations with survival outcomes in the multivariate analysis (*p* > 0.05; Figure 6 and Figure 7).

### 3.5. In Silico Validation of Autophagy-Related Gene Expressions

**Figure 8:**
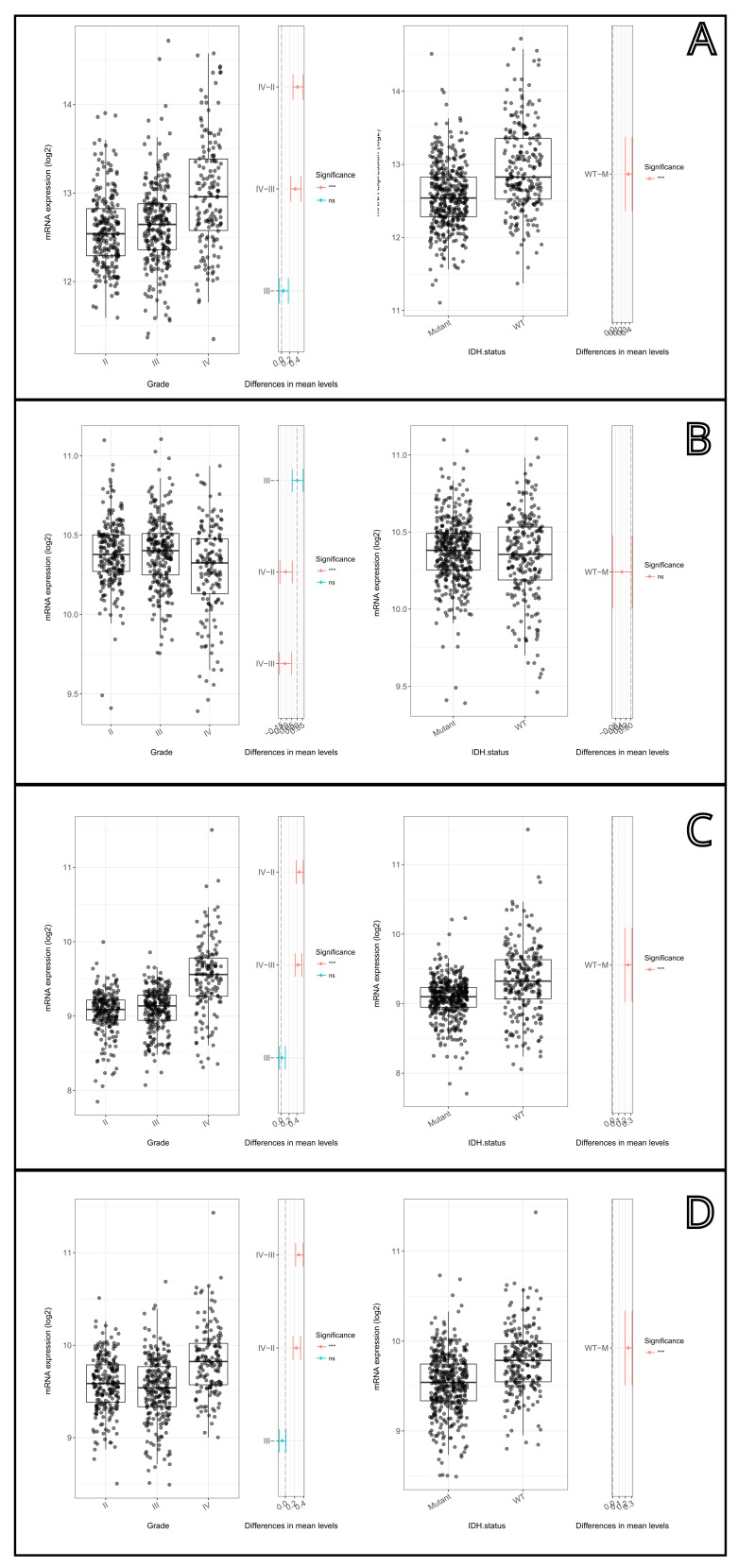
In silico validation of autophagy-related gene ((**A**) SQSTM1, (**B**) BECN1, (**C**) ATG5, and (**D**) ATG7) mRNA expression levels using an independent glioma dataset via the GlioVis data portal. Left panels illustrate differential expression across WHO tumor grades (Grades II, III, and IV). Right panels display the comparison of gene expression profiles based on IDH mutation status (Mutant vs. Wild-Type). Statistical significance is denoted by asterisks (***), which indicate a highly significant difference in overall survival (*p* < 0.001), whereas the absence of it represents a non-significant result.

To validate the robustness of our 4-gene panel and address its prognostic significance in a larger, independent cohort, we performed an in-silico analysis using the GlioVis data portal (Dataset: TCGA_GBMLGG). Consistent with our qRT-PCR findings, the independent dataset revealed significant differential expression of the targeted autophagy genes across varying tumor grades and IDH mutation statuses (Figure 8).

**Figure 9:**
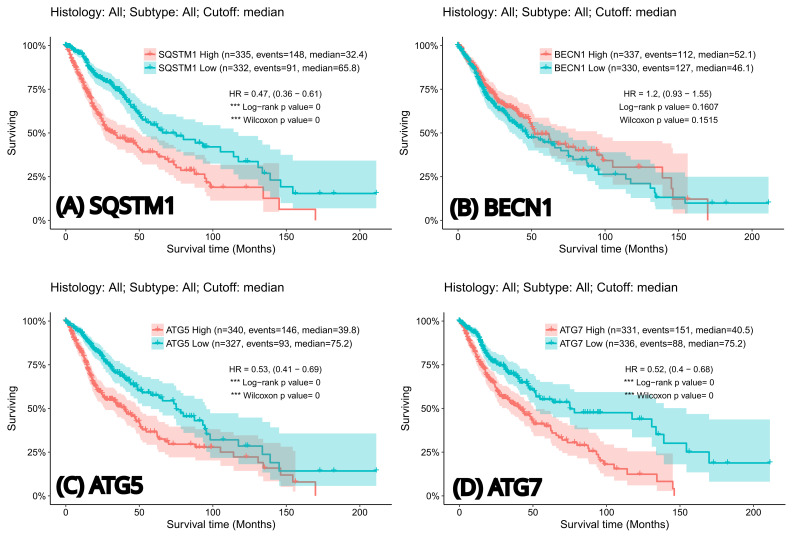
Kaplan-Meier survival curves generated from an independent cohort via the GlioVis platform, evaluating the prognostic impact of autophagy-related genes. The plots demonstrate the association between high (red) versus low (blue) mRNA expression levels of (**A**) SQSTM1, (**B**) BECN1, (**C**) ATG5, and (**D**) ATG7 and overall survival (OS) in glioma patients. Statistical significance is denoted by asterisks (***), which indicate a highly significant difference in overall survival (*p* < 0.001), whereas the absence of it represents a non-significant result. The Hazard Ratio (HR) describes the relative risk, where values less than 1 indicate a higher risk for the “High” expression group, while the Log-rank and Wilcoxon tests serve as the statistical measures used to compare the survival distributions between the groups.

Notably, Kaplan-Meier survival analysis of the validation cohort strongly corroborated our primary findings: high expression of SQSTM1, Atg5, and Atg7 were significantly associated with poorer survival outcomes (*p* < 0.05), further solidifying their roles as aggressive tumor markers (Figure 9). Although Beclin1 did not reach strict statistical significance for survival in this specific in silico cohort (*p* = 0.1607), its mechanistic importance in IDH-mutant tumors remains evident.

## 4. Discussion

In this research, the expression levels of autophagy-related genes SQSTM1, Beclin1, Atg5, and Atg7 in diffuse astrocytic tumors were evaluated, and the relationship of these genes with tumor grade, IDH mutation status, and survival parameters were explored. The findings obtained indicate that the autophagy mechanisms may have a critical role in glioma biology and tumor cell survival under stress conditions [5,15]. In our study, a significant relation between WHO tumor grade and autophagy gene expressions was not detected. However, there are different findings in the literature concerning the relation between autophagy protein expression and tumor grade; some papers have reported increased expression in high-grade tumors [11], whereas others suggest more sustainable autophagy activity in low-grade gliomas [12]. In our study, in the analysis according to the IDH mutation status, it was found that the gene expressions of Beclin1 and Atg5 were significantly different and the ΔCt values of these genes were lower in IDH mutant cases (thus the expression was higher). This finding is consistent with the recent data showing that the oncometabolite 2-hydroxyglutarate (2-HG), which accumulates as a result of IDH mutation, can induce autophagy pathways in glioma cells through Beclin1 and LC3B activation [14,28]. Furthermore, it has been found that the SQSTM1 (p62) gene expression is independently related to both overall survival and progression-free survival. In the literature, the accumulation of p62 is also strongly associated with poor prognosis and shortened survival times in advanced-stage tumors [11,20,29]. These findings support that autophagy-related genes may serve as potential biomarkers for determination of glioma prognosis and development of novel therapeutic strategies [3,11].

Gliomas are the most common primary malignant tumors of the central nervous system and account for approximately 80% of all malignant brain tumors; their clinical courses are largely dependent on tumor grade and molecular features [30]. Particularly WHO tumor grade and patient age are considered the most basic and critical factors determining prognosis and survival time in glioma patients [30,31,32]. In our study, the identification of age and tumor grade as independent prognostic factors for both overall survival and progression-free survival is in complete agreement with the common findings in the literature [31,32]. However, in recent years, the molecular mechanisms have become increasingly important in understanding glioma biology; especially, it has been shown that adaptation of tumor cells to stress conditions through autophagy pathways governs tumor development, invasion capacity, and resistance to treatment [12,15,19].

Autophagy is a process where cells clear out damaged proteins and organelles that are wrapped in a double membrane structure called autophagosome which fuse with lysosome degrading the contents. It is a central cellular mechanism that has been evolutionarily conserved and was extensively discussed by Ichimura & Komatsu (2011) [33], Mizushima (2011) [34]. Among various extremes of cellular environment, this mechanism plays a major role helping the cell to save energy and maintain homeostasis especially in the case of metabolic stress factors such as hypoxia, nutrient deprivation and oxidative stress [34,35,36]. Autophagy has been found to play different and “double-edged sword” roles in cancer biology depending on cellular context and microenvironment conditions [3,37]. The major role of autophagy in tumor biology may be autophagy at the early stages of tumor development by unloading damaged organelles and oncogenic molecules, maintaining genomic stability and reducing oxidative stress thereby acting as a tumor suppressive mechanism [35]. However, with tumor advancement, especially in the late stages, the same mechanism supports tumor cell survival under metabolic stress and unfavorable microenvironment conditions, nutrient recycling as well as developing resistance to therapy that in turn leads to tumor progression and aggressiveness [38,39].

In the case of high-grade gliomas such as Glioblastoma, the tumor microenvironment is profoundly hypoxic and metabolically stressed due to inadequate vascularization [15,25]. These harsh conditions through the activation of autophagic machinery, for example, via pathways involving HIF-1α and BNIP3, help tumor cells in metabolic adaptation of the environment [10,23,25]. Through autophagy, the lysosomal degradation of damaged organelles and proteins is performed, and cellular components recycling is achieved; the metabolic substrates that include amino acids and fatty acids obtained from this process help the cell to maintain its energy homeostasis [10,15,19]. This contributes directly to tumor cells’ ability to survive under nutrient deprivation and hypoxia conditions in the tumor microenvironment, resistance development, and phenotypic changes towards more aggressive behavior [23]. Therefore, it is recognized that autophagy machinery plays a very important role in tumor cell adaptation, survival, and drug resistance, particularly in aggressive gliomas [15,18,29].

We determined that SQSTM1, Beclin1, Atg5 and Atg7 gene expressions were not significantly different among the WHO tumor grades in our study. Although few studies in the literature report that autophagy markers show higher expression in high grade gliomas [11,40], some other studies mention that these markers are not directly associated with tumor grade or low-grade gliomas may have more sustained autophagy activity [12,40]. This implies that the autophagy mechanism cannot be explained solely by the histopathological grade and is regulated dynamically by many complex factors such as tumor microenvironment, metabolic reprogramming, hypoxia levels and specific molecular genetic changes [15,23,41].

One of the critical findings of our study was the association of Beclin1 and Atg5 gene expressions with IDH mutation status. IDH mutations, which play a crucial role in the molecular classification of gliomas, are usually associated with better prognosis and different metabolic profile [42,43]. Mutations in IDH1 and IDH2 genes cause a dramatic change in cellular metabolism leading to the accumulation of the oncometabolite 2-hydroxyglutarate (2-HG) [44,45]. Accumulation of 2-HG results in inhibition of α-KG-dependent dioxygenases leading to DNA and histone hypermethylation, and this, in turn, contributes to reprogramming of cellular metabolic pathways and gene expression profiles [46,47].

Our finding that the expression of Beclin1 and Atg5 was higher in IDH mutant tumors as compared to IDH wild-type tumors (based on the ΔCt values showing IDH mutant cases having significantly lower values) denotes that these genes were highly expressed in mutant IDH glioma cells. The observation in various studies supporting 2-HG inducing autophagy via LC3B activation and Beclin1 expression, and that this mechanism is vital for the survival of IDH mutant cells under metabolic stress, also explains our findings [14,15,28]. Furthermore, the fact that SQSTM1 (p62) gene has an independent association with survival is in line with the current view that p62 accumulation is a strong biomarker for glioma progression and bad prognosis [11,20,29]. Interestingly, while both Atg5 and Atg7 are essential for autophagosome formation, only Atg5 showed a significant expression difference correlated with IDH mutation status in our cohort. This divergence can be explained by the broader, non-canonical functions of Atg5. Unlike Atg7, which functions strictly as an E1-like activating enzyme, Atg5 actively participates in apoptotic cross-talk and can be cleaved to induce cell death pathways [48]. This suggests that the metabolic reprogramming induced by 2-HG accumulation might selectively target and exploit Atg5-dependent pathways to finely tune the balance between cell survival and death under extreme stress.

Metabolic reprogramming can also directly influence the regulation of autophagy activity through the AMPK, mTOR axis. Changes in energy metabolism of glioma cells, by triggering activation of AMPK as energy sensor, lead to inhibition of the mTORC1 complex and consequently modulation of autophagy processes [10,49]. In this mechanism, Beclin-1 is one of the key components of class III PI3K complex that is necessary for the initiation of autophagosome formation [19]. Interaction of Beclin-1 with anti-apoptotic proteins from the Bcl-2 family is considered as a crucial molecular switch determining the balance between autophagy and apoptosis. Beyond initiating autophagy, the dynamic dissociation of Beclin1 from Bcl-2 allows tumor cells to survive harsh microenvironments through protective autophagy, while excessive free Beclin1 can disrupt apoptotic regulation [16,19]. In the context of our findings, the heightened Beclin1 expression in IDH-mutant tumors may reflect an active attempt by these cells to evade apoptosis and sustain survival via autophagic induction under severe metabolic stress.

In IDH mutant gliomas, the metabolic profile altered due to the accumulation of the oncometabolite 2-hydroxyglutarate (2-HG) can cause the autophagy activity to be regulated at different levels, thereby affecting the expression of autophagy initiating proteins such as Beclin-1 and LC3B [14,28]. In our study, the expression of Beclin-1 was found to be associated with the IDH mutation status and higher expression was observed in mutant cases, which is in line with recent results indicating that 2-HG may induce the autophagic pathway. This supports a very strong interaction between glioma metabolism and autophagy mechanisms [15,28].

Another major finding from our work is the direct relationship between SQSTM1 gene expression and survival parameters. SQSTM1 protein, also known as p62, is a critical adaptor protein that contributes to the autophagic degradative pathway by directing ubiquitinated proteins to autophagosomes [29,50]. Besides this, it has been shown that the p62 protein, by regulating onco-signaling pathways such as NF- κB, mTORC1 and κ1-Nrf2, contributes to tang tumor cells proliferation, survival and resistance to therapy [50,51]. Recent glioblastoma papers report that high p62 expression and cytoplasmic accumulation are strongly associated with increased tumor aggressiveness, mesenchymal transition and poor prognosis [20,29,51].

The autophagy mechanism has been shown to dynamically affect not only tumor cells but also tumor microenvironment. Recent studies have demonstrated that autophagy is key to immune cell infiltration, metabolic interactions with stromal cells and behavior of glioma stem cells [3,40,41]. The dependence of glioma stem cells on autophagy machinery to maintain their pluripotency and for survival during metabolic stress conditions such as hypoxia and nutrient deprivation has been particularly highlighted [41,52,53].

For this reason, autophagy pathways are primarily considered among the potential therapeutic targets for glioblastoma treatment. Especially the use of autophagy inhibitors such as chloroquine and hydroxychloroquine together with chemotherapy (temozolomide) and radiotherapy, is being investigated in clinical trials as a potential strategy to sensitize tumor cells to treatment and extend median survival times [54,55]. Pharmacological inhibition of autophagy has the potential to decrease the development of resistance to therapy by disrupting the metabolic adaptation capacity of tumor cells and repressing stemness features [52,56].

This study has some strong points. Firstly, the simultaneous evaluation of multiple autophagy-related genes in the same cohort of diffuse astrocytomas has helped thoroughly investigate the role of autophagy pathways in glioma biology. Besides, the integration of gene expression data with clinical parameters and survival analyses enhances the clinical significance of the study. However, there are some limitations to the study. First of all, the study has a retrospective design and the gene expression analyses have been carried out only at the mRNA level. Since autophagy activity is regulated at the protein level and through functional mechanisms, data only at the transcript level may limit the full assessment of autophagic activity. Furthermore, the study was carried out in a single center, and the limited number of patients may restrict the generalizability of the findings. Multicenter studies and validation analyses at the protein level in future will shed more light on the role of autophagy-related genes in glioma prognosis. Although our findings were strengthened by independent in silico validation, confirming the prognostic robustness of this 4-gene panel, the mechanistic details of these clinical associations remain to be fully elucidated. Future comprehensive in vitro and in vivo functional analyses are strictly required to unmask the exact molecular mechanisms by which these targeted autophagy genes drive tumor progression and therapy resistance.

## 5. Conclusions

In summary, our study demonstrates that autophagy-related genes, especially SQSTM1 and Beclin1, could be associated with tumor biology and survival in diffuse astrocytic tumors. Besides, the findings that Beclin1 and Atg5 gene expressions are correlated with the IDH mutation status may represent the possible interaction between glioma metabolism and autophagy mechanisms. These findings independently demonstrate that autophagy pathways could be used as potential biomarkers in the assessment of glioma prognosis and, at the same time, represent a very important research area for the development of targeted therapeutic strategies.

## Figures and Tables

**Table 1:** Baseline Demographic and Clinicopathological Characteristics of the Study Cohort.

Characteristic	Value
Total Patients	150
Age (Years, Mean ± SD)	55.1 ± 14.1
**Gender, n (%)**	
Male	94 (62.7%)
Female	56 (37.3%)
**WHO 2021 Tumor Grade, n (%)**	
Grade 2	20 (13.3%)
Grade 3	33 (22.0%)
Grade 4 (Glioblastoma)	97 (64.7%)
**IDH1 R132H Mutation Status, n (%)**	
Mutant	70 (46.7%)
Wild-type	80 (53.3%)

**Table 2:** Primer sequences for qPCR analysis.

Gene	Forward Primer (5′–3′)	Reverse Primer (5′–3′)	**Ref.**
*SQSTM1*	AGGCGCACTACCGCGAT	CGTCACTGGAAAAGGCAACC	[24]
*BECN1*	GGGGATCCGGAAGTTTTCGGCGGCTA	GGGGGAATTCGAAGAAAGGGAAAGGAGT	[25]
*ATG5*	CAGTTTGGCACACACTTGTG	GTCTGTGATGGGTTGTTGCT	[26]
*ATG7*	ACACCAAGAGGAGCTGTTGA	TGTGCTGTTGCTGTAGGTGT	[27]

## Data Availability

Data that is used in this manuscript is available upon request from the corresponding author.

## Abbreviations

The following abbreviations are used in this manuscript:

ARGs	Autophagy-Related Genes
Atg5	Autophagy Related 5
Atg7	Autophagy Related 7
BECN1	Beclin 1
CNS	Central Nervous System
Ct	Cycle Threshold
FFPE	Formalin-Fixed Paraffin-Embedded
GBM	Glioblastoma
H&E	Hematoxylin and Eosin
HIF-1	Hypoxia-Inducible Factor 1
IDH	Isocitrate Dehydrogenase
IHC	Immunohistochemistry
OS	Overall Survival
PFS	Progression-Free Survival
qRT-PCR	Quantitative Real-Time Polymerase Chain Reaction
SQSTM1	Sequestosome 1
WHO	World Health Organization
